# Establishment of integrated protocols for automated high throughput kinetic chlorophyll fluorescence analyses

**DOI:** 10.1186/s13007-017-0204-4

**Published:** 2017-07-04

**Authors:** Henning Tschiersch, Astrid Junker, Rhonda C. Meyer, Thomas Altmann

**Affiliations:** Leibniz Institute of Plant Genetics and Crop Plant Research (IPK) Gatersleben, Corrensstr. 3, 06466 Seeland, OT Gatersleben, Germany

**Keywords:** Chlorophyll fluorescence imaging (CFI), High throughput screening, Plant phenotyping, Photosynthesis, PSII operating efficiency

## Abstract

**Background:**

Automated plant phenotyping has been established as a powerful new tool in studying plant growth, development and response to various types of biotic or abiotic stressors. Respective facilities mainly apply non-invasive imaging based methods, which enable the continuous quantification of the dynamics of plant growth and physiology during developmental progression. However, especially for plants of larger size, integrative, automated and high throughput measurements of complex physiological parameters such as photosystem II efficiency determined through kinetic chlorophyll fluorescence analysis remain a challenge.

**Results:**

We present the technical installations and the establishment of experimental procedures that allow the integrated high throughput imaging of all commonly determined PSII parameters for small and large plants using kinetic chlorophyll fluorescence imaging systems (FluorCam, PSI) integrated into automated phenotyping facilities (Scanalyzer, LemnaTec). Besides determination of the maximum PSII efficiency, we focused on implementation of high throughput amenable protocols recording PSII operating efficiency (Φ_PSII_). Using the presented setup, this parameter is shown to be reproducibly measured in differently sized plants despite the corresponding variation in distance between plants and light source that caused small differences in incident light intensity. Values of Φ_PSII_ obtained with the automated chlorophyll fluorescence imaging setup correlated very well with conventionally determined data using a spot-measuring chlorophyll fluorometer. The established high throughput operating protocols enable the screening of up to 1080 small and 184 large plants per hour, respectively. The application of the implemented high throughput protocols is demonstrated in screening experiments performed with large Arabidopsis and maize populations assessing natural variation in PSII efficiency.

**Conclusions:**

The incorporation of imaging systems suitable for kinetic chlorophyll fluorescence analysis leads to a substantial extension of the feature spectrum to be assessed in the presented high throughput automated plant phenotyping platforms, thus enabling the simultaneous assessment of plant architectural and biomass-related traits and their relations to physiological features such as PSII operating efficiency. The implemented high throughput protocols are applicable to a broad spectrum of model and crop plants of different sizes (up to 1.80 m height) and architectures. The deeper understanding of the relation of plant architecture, biomass formation and photosynthetic efficiency has a great potential with respect to crop and yield improvement strategies.

## Background

Plant phenotyping is an emerging area of science acquiring plant traits, especially those relevant for biomass formation and yield, for resistance to stresses, and for resource efficiency, in an automated, non-invasive and high throughput manner. This enables the association of these important features of plants to genomic information in order to identify genetic components underlying trait expression. In this context it is obvious that successful crop improvement strategies rely on the integrated assessment of genomic and phenomic data with the latter comprising a comprehensive set of plant traits accurately quantified in large plant populations, as an essential prerequisite for linkage mapping or genome-wide association mapping of quantitative trait loci (QTL).

During the last few years, a number of high throughput (HT) screening technologies have become available and respective facilities have been set up for high throughput phenotyping of model and crop plants, many of them though mainly focussing on the quantification of plant architectural and biomass-related traits [1–4]. However, the biomass component of the yield equation largely depends on radiation use efficiency, which in turn is influenced by both morphology (canopy architecture, light interception) and photosynthetic performance (light use efficiency) of plants [5]. In terms of crop (yield) improvement, this illustrates the need for integrated solutions for the simultaneous assessment of plant morphology and physiology (photosynthesis) and the implementation of methods for the quantitative measurement of the physiological state of plants in high throughput [6]. PSII fluorescence emission is especially suited to monitor physiological traits via changes in photochemistry [7]. Chlorophyll fluorescence imaging (CFI) represents a non-destructive method that can be applied repeatedly during plant growth. It allows the time resolved two-dimensional analysis of chlorophyll fluorescence transients on the basis of images on the leaf- or whole-plant level [8]. Moreover, as a non-invasive and comparatively rapid technique with measurement times of minutes down to seconds per plant on a whole-shoot level it is well suited for HT automated imaging [5, 6, 9].

Most modern fluorometers use a modulated light source. The application of this pulse-amplitude modulated (PAM) technique allows the analysis of activity and regulation of photosystem II and provides information about the integrity status of the photosynthetic apparatus. Commonly, plants are treated with light of known frequency (modulated) to induce chlorophyll fluorescence and the detector is set to measure at the same frequency [10]. The result is not influenced by ambient light and it is a clear advantage that the measurements can be performed without darkening of the plants. The technique furthermore involves the application of a very bright saturating flash of light that temporarily leads to a maximal plastoquinone A (Q_A_) reduction and therefore closes all PSII reaction centres [11, 12]. Therefore this method allows the separation of photochemical and non-photochemical fluorescence quenching processes [6] and the calculation of the photosystem II operating efficiency (Φ_PSII_). Among a variety of commonly used fluorescence parameters, Φ_PSII_ is of special relevance as a suitable proxy for light-use efficiency [13]. It is a measure of the relative quantum yield of PSII (the proportion of absorbed light that is actually used in photochemistry of PSII) and therefore can be used for the calculation of the linear electron transport rate [14]. This process is clearly correlated to the quantum yield of the CO_2_ assimilation and the overall photosynthetic capacity [15]. Measurements of Φ_PSII_ enable rapid screening of large numbers of plants [16, 17], which in this way can be monitored for their photosynthetic performance at nearly the same time and under almost identical conditions when cultivated in controlled environments.

A number of facilities have been implemented in the past years which apply CFI for medium to high throughput investigation of small rosette plants (Fluor*Imager* [18]; GROWSCREEN FLUORO [19]; Phenovator [20]; PlantScreen system [21]). More recently, some platforms for medium throughput analysis of medium-sized plants (such as cereals up to 1.40 m) have been set up (National Plant Phenotyping Infrastructure, NaPPI, Helsinki, Finland, http://blogs.helsinki.fi/nappi-blog/highthroughput/; Crop Plant Shoot Module at the High Resolution Plant Phenotyping Centre, HRPPC, Canberra, Australia, http://www.plantphenomics.org.au/services/cropshoot/; Bellwether Foundation Phenotyping Facility, St. Louis, USA, https://www.danforthcenter.org/scientists-research/core-technologies/phenotyping and several PlantScreen™ phenotyping systems at DGIST, Daegu, South Korea, at the Center of the Region Haná for Biotechnological and Agricultural Research, China; Olomouc, Czech Republic and at IBERS, Aberystwyth, UK). Facilities for HT CFI in large (crop) plants (>1.50 m plant height) have hitherto not been reported although its need is highlighted by the huge number of potential applications with high relevance for crop improvement and plant breeding. CFI has a wide range of applications to diagnose the reaction of plants to biotic and abiotic stresses even before visible symptoms become apparent, thereby representing a fast screening method for genotypes with increased resistance (for review see [22]). A number of publications describe the use of CFI in studies on drought stress [19, 23, 24], chilling/cold stress [19, 25–27] and nutrient deficiency [28]. Several other studies applied CFI for the early identification of genotypes with high tolerance to biotic stress [29–32] and for the analysis of acclimation processes in response to light treatments [33]. In the context of strategies for crop yield increases the process of photosynthesis, as an important factor underlying plants’ light use efficiency, bears substantial potential for improvement [34] which motivated several studies on the analysis of natural variation in photosynthetic characteristics and its potential relations to yield [6, 13, 35].

Here we describe the integration of CFI systems into existing HT plant phenotyping facilities, enabling the integrated assessment of plant architectural and biomass related parameters as well as physiological features such as photosynthetic efficiency in plants up to 1.80 m height. Protocols for the fast measurement of chlorophyll fluorescence parameters have been implemented and validated on the basis of comparative manual measurements with standard low throughput devices. The presented uses of the systems to screen large panels of Arabidopsis and maize accessions for variation in PSII operating efficiency reveal the performance of the automated HT CFI systems. The potential of the presented integrated approach in terms of crop yield improvement and its relevance for breeding strategies is discussed.

## Results

### Technical properties of the integrated chlorophyll fluorescence imaging systems

**Fig. 1:**
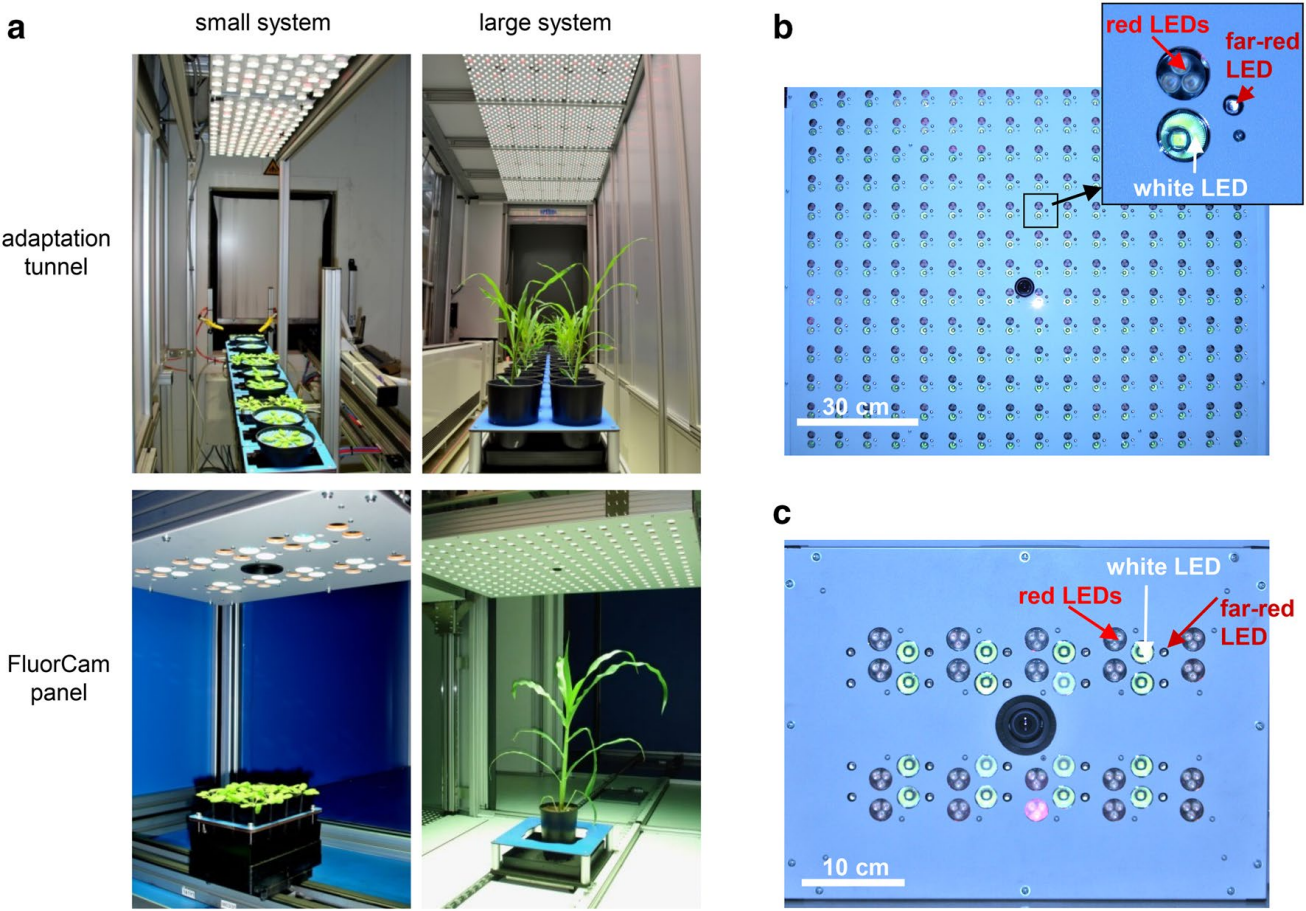
FluorCam-systems integrated in LemnaTec Scanalyzer systems for small and large plants, respectively. **a** FluorCam-panels (operating in *top view* orientation) and adaptation tunnels. **b** Arrangement of LEDs across the FluorCam panel of the large system. **c** Arrangement of LEDs across the FluorCam panel of the small system

**Fig. 2:**
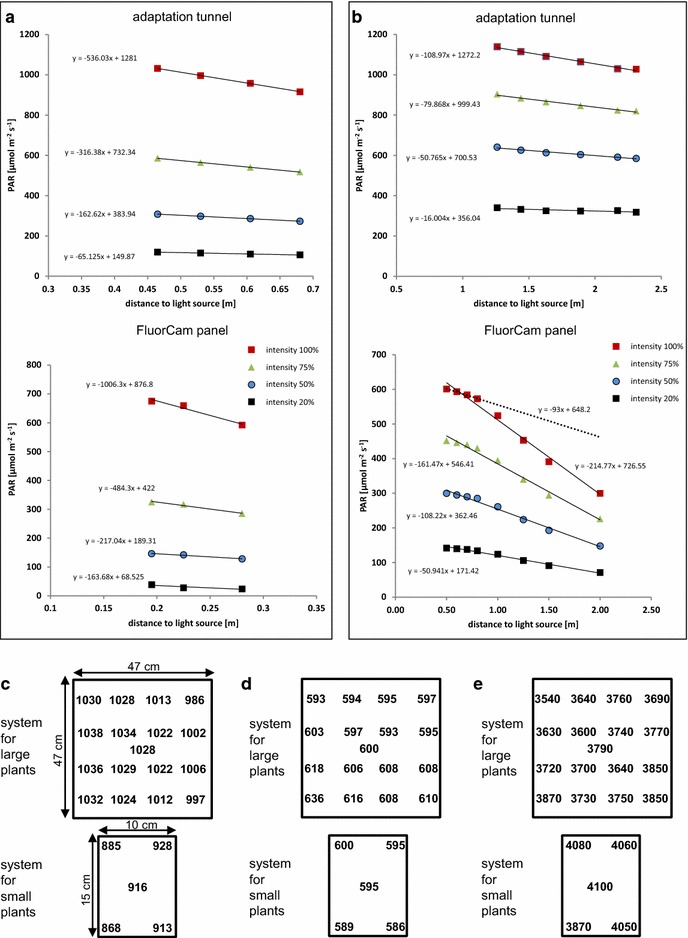
Light intensity distribution in FluorCam systems. Measured light intensities in relation to the distance of the FluorCam panel (white emitting diodes) or of the light panels installed in the adaptation tunnel, respectively. Different light intensities (given in %) were tested, while the distance of the micro-quantum sensor was varied. **a** System for small plants. **b** System for large plants. **c**–**e** Spatial distribution of PAR intensities across the carrier top area. **c** LED panel derived light intensity in adaptation tunnels (at maximum setting, measured at carrier level). **d** FluorCam panel derived actinic light intensities (at maximum setting, measured at working distance). **e** Intensity of FluorCam panel derived saturating pulse (at maximum setting, measured at working distance). All shown values of PAR are given in µmol m^−2^ s^−1^

Three different Scanalyzer systems (LemnaTec AG, Aachen, Germany) for automated high throughput cultivation and imaging of plants with various dimensions are available at the IPK Gatersleben. The system for small plants is situated in a growth chamber and allows the non-destructive trait assessment of up to 4600 Arabidopsis plants. The system for large plants has a capacity of up to 1684 large plants and is placed in a glasshouse chamber. Detailed and standardized protocols for plant cultivation adapted to the special requirements of the high throughput plant phenotyping approaches using these Scanalyzer systems have been described previously [4]. Both systems have been upgraded with FluorCam devices for imaging-based kinetic chlorophyll fluorescence analyses (Photon Systems Instruments (PSI), Brno, Czech Republic). The FluorCam devices work with weak modulated measuring light in combination with saturating light flashes and actinic light (light that drives photosynthetic electron transport) according to the PAM-principle and thereby enable the separation of photochemical and non-photochemical fluorescence quenching processes. All common fluorescence parameters (see Table 1) can be recorded including maximum quantum efficiency of PSII (F_v_/F_m_), PSII operating efficiency (Φ_PSII_), electron transport rate (ETR) and the degree of non-photochemical fluorescence quenching (NPQ). The FluorCam installations consist of two main modules: a light adaptation tunnel and a FluorCam imaging module (Fig. 1a). The latter consists of a CCD camera and surrounding panel of light-emitting diodes (LEDs) which generate the measuring light, the actinic light and the saturating flashes (Fig. 1b, c). The panel contains diodes providing cool white light (broad emission peak with wavelength in the range 480–720 nm and an additional sharp emission peak at 436 nm) and additionally diodes for orange-red with a peak emission wavelength of 620 nm (half width approximately 5 nm) and diodes with a peak emission in the far-red spectral region (sharp peak at 735 nm, half width approximately 12 nm). The white and red LEDs are equipped with collimators resulting in a narrow emission angle of approx. 13° (white LEDs) or 16° (red LEDs), respectively. This results in a low and nearly linear vertical decline of the light intensity (see below and Table 2) and thereby guarantees a high homogeneity of PAR across the measured area (Fig. 2c–e). The CCD camera is highly sensitive and captures the fluorescence images with a resolution of 720 × 560 effective pixels.

**Table 1:** Chlorophyll fluorescence parameters measurable with FluorCam systems (following nomenclature in [6] or [36] regarding Rfd, respectively)

Fluorescence parameter	Definition
F, F′	Steady state fluorescence emission from dark- or light-adapted leaf, respectively
F_0_, F_0_′	Minimal chlorophyll fluorescence intensity measured in the dark- or light-adapted state, respectively
F_m_, F_m_′	Maximal chlorophyll fluorescence intensity measured in the dark- or light-adapted state, respectively
F_v_, F_v_′	Variable chlorophyll fluorescence (F_m_-F_0_) measured in the dark- or light-adapted state, respectively
F_q_′	Difference in fluorescence between F_m_′ and F′
F_v_/F_m_	Maximum quantum yield of PSII photochemistry measured in the dark-adapted state
F_p_	Peak fluorescence during the initial phase of the Kautsky effect
Rfd	Fluorescence decline ratio in steady-state (F_p_ − F′)/F′
Φ_PSII_	PS II operating efficiency; effective quantum yield of photochemical energy conversion in PSII (F_q_′/F_m_′)
NPQ	Non-photochemical quenching (F_m_/F_m_′) – 1
qL	Fraction of PSII centers that are ‘open’ based on the lake model of PSII (F_q_′/F_v_′)(F_0_′/F′)
ETR	Electron transport rate

**Table 2:** Technical light parameters of LED-panels

	Small plant system	Large plant system
*LED panel adaptation tunnel*		
Max. PAR at 100% light intensity [µmol m^−2^ s^−1^] measured at carrier level	916	1028
Decrease in light intensity per cm distance to light source measured for 100% light intensity	5 µmol m^−2^ s^−1^	1 µmol m^−2^ s^−1^
*FluorCam-panel (white LEDs)*		
Max. PAR at 100% light intensity [µmol m^−2^ s^−1^] measured at working distance	595	600
Decrease in light intensity per cm distance to light source measured for 100% light intensity	10 µmol m^−2^ s^−1^	0.9–2.3 µmol m^−2^ s^−1^

The FluorCam modules are installed inside of imaging chambers thus avoiding environmental variation of light during measurements. The panel for large plants can be adjusted in height dependent on the average height of the plants to be measured. Both FluorCam systems enable top view imaging of plants, whereas the system for small plants provides the additional opportunity for side view imaging. Thus, the latter system allows the analysis of rosette plants such as Arabidopsis (top view) and plants with a height up to 10 cm such as young seedlings of different plant species (side view). The imaging area in top view position is 110 × 140 mm. The system for large plants is suitable for imaging of plants with a height up to 1.80 m (such as maize, wheat or rapeseed) and has an imaging area in top view position of 800 × 800 mm. The saturating pulses (measured in the centre of the plant transport carrier) have light intensities of 4100 and 3800 µmol m^−2^ s^−1^ in the systems for small and large plants, respectively. Multiple measurements of PAR intensity (Fig. 2e) demonstrated a good homogeneity of the intensity distribution across the plant transport carrier area (3540–3870 µmol m^−2^ s^−1^ in the system for large plants and 3870–4100 µmol m^−2^ s^−1^ in the system for small plants, respectively).

Adaptation tunnels guarantee equal light exposure of the plants prior to the recording of fluorescence data and therefore are an essential prerequisite for the precise and accurate measurement, especially in case of Φ_PSII_. The adaptation tunnels are equipped with LED-panels comprised of diodes producing cool white light and additionally diodes for orange-red and far-red light (as already described for the FluorCam-panel) in order to provide a balanced light spectrum. The major portion of PAR is produced by white LEDs, approximately 5% originates from orange-red LEDs and 0.4% from far-red LEDs, respectively. The adaptation area provides space for simultaneous adaptation of up to 132 plants (12 per carrier) and 40 plants (4 per carrier) with maximum light intensities (measured in the centre of the carrier) of 916 and 1028 µmol m^−2^ s^−1^ photosynthetic active radiation (PAR) in the systems for small and large plants, respectively. Multiple measurements performed in order to characterize variability of PAR levels across the carrier area illustrate the homogeneity of illumination [range of PAR intensity: 868–928 µmol m^−2^ s^−1^ in the small system and 986–1038 µmol m^−2^ s^−1^ in the large system, respectively (Fig. 2c)].

After their capture, the acquired raw fluorescence images are processed using automated image analysis routines which are implemented in the FluorCam software package (http://psi.cz/downloads/). Following a segmentation step for the definition of the area of interest (the plant), this software routinely performs a single-pixel based fluorescence feature extraction. Alternatively, the calculation with averaged mean values for the complete area of interest (the whole plant) is possible in a manual mode. For the analysis of multiple plants within a single image (as for multi-well trays with 12/6 or 4/2 plants per carrier in the small and large plant systems, respectively) pre-defined masks enable the selection of several areas of interest. All raw/processed images and the extracted trait values in tabular format (csv) are stored in a cloud based database (DB) system using a compressed data archive format. The result data file includes unique plant IDs thereby allowing the integration of the FluorCam-derived data with phenotypic values detected with other devices of the systems.

The assessment of photosynthetic parameters in entire plant shoots is challenging compared to measurements on a single leaf or leaf spot level. Especially gradients in effective light intensity and shading effects caused by the architecture of the plants may influence the analysis of fluorescence data and complicate data interpretation. Therefore we paid special attention to light conditions (intensity distributions) in the adaptation areas and inside the CFI chambers. Manual PAR measurements demonstrated that light intensities decreased almost linearly and in a shallow gradient with increasing distance to the light source in the imaging chambers as well as adaptation tunnels of both systems. In the adaptation areas, the effective light intensities were found to decrease by about 5 and 1 µmol m^−2^ s^−1^ in the systems for small and large plants, respectively, with every cm distance to the light source when set to the maximum intensities (Fig. 2; Table 2). The incident intensity of the FluorCam panel emitted light in the system for small plants decreased by 10 µmol m^−2^ s^−1^ per cm (measured for the maximum light intensity, Fig. 2a). For the large FluorCam panel it was found that the decrease in light intensity within distances to the light sources of about 50–80 cm (see dotted line in Fig. 2b) is lower (0.9 µmol m^−2^ s^−1^ per cm) compared to distances above 80 cm (2.3 µmol m^−2^ s^−1^ per cm). For a working PAR of 350 µmol m^−2^ s^−1^ (~55% of the FluorCam panel maximum intensity) which corresponds to the average amount of PAR in the plant growth area of the greenhouse in the system for large plants, the incident light intensity decreased by only 1 µmol m^−2^ s^−1^ per cm distance to the light source (Fig. 2b). Based on these findings, for all measurements the light intensity settings were always adjusted to reach the desired amount of PAR in the recommended working distance of about 27 and 55 cm in systems for small and large plants, respectively. The described shallow linear decrease of the amount of PAR with increasing distance to the light source is achieved by the dimension and the design of the light panels which consist of focussed LEDs (equipped with collimators) in high density. The FluorCam panel of the large system (size: 120 cm × 120 cm) incorporates 224 white and far-red LEDs and 672 orange-red LEDs (Fig. 1b). The panel of the small system (size: 47 cm × 34 cm) contains 20 white and far-red LEDs and 60 orange-red LEDs (Fig. 1c). Due to this setup light intensities are not inversely proportional to the square of the distance from the light source.

### Influence of the incident light intensity on Φ_PSII_ determination

**Fig. 3:**
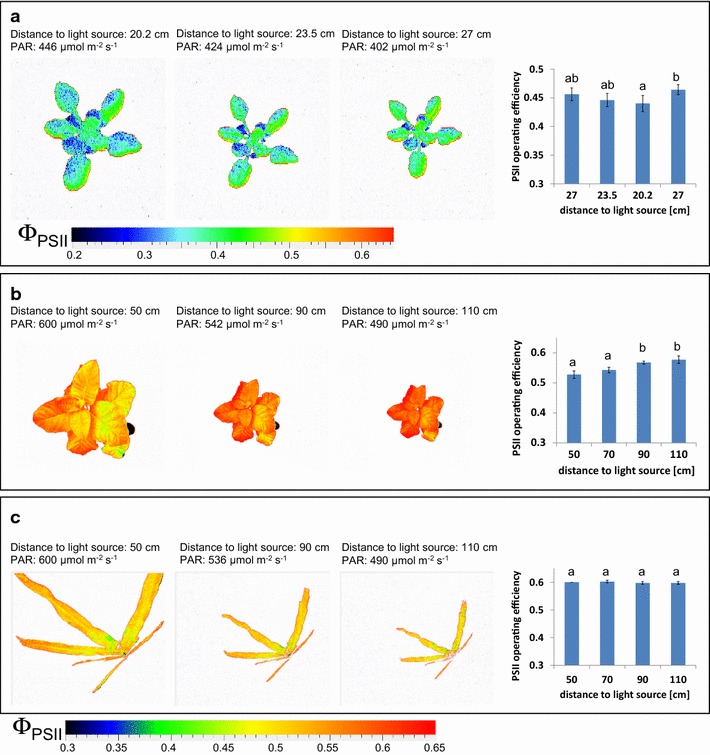
Changes in incident light intensity caused by variation of the distance between FluorCam panels and plants: influence on Φ_PSII_ determination. False *colour images* were captured for **a** Arabidopsis Col-0 plants (28 DAS) using the system for small plants or **b** *Nicotiana tabacum SNN* (35 DAS) and **c** *Zea mays* (36 DAS) using the system for large plants. Light intensities were measured as indicated. The data are the mean ± SD of 4–5 replicate measurements. *Letters* indicate statistically significant differences at the *p* < 0.05 level according to ANOVA and post-hoc Bonferroni test

**Fig. 4:**
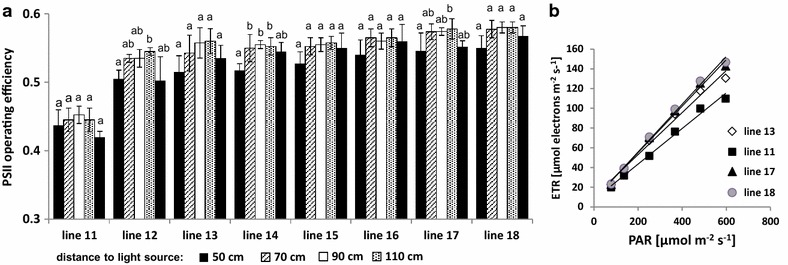
Effect of changes in incident light intensities on the amount of Φ_PSII_ in different maize accessions (27 DAS). **a** Influence of distance variation between FluorCam panels and plants on the determination of Φ_PSII_ (n = 4–5). The values of light intensities at different distances to light sources are given in Fig. 3. *Letters* indicate statistically significant differences at the *p* < 0.05 level according to ANOVA and post-hoc Bonferroni test. **b** Light-response curves of apparent electron transport rates (ETR) measured with the FluorCam system for large plants. Actinic light was applied during consecutive 60 s periods with stepwise decreasing intensity. The data are the mean ± SD of 4–5 replicate measurements

In order to assess the influence of the determined variation in incident light intensity on fluorescence parameters, Φ_PSII_ was measured in Arabidopsis, tobacco and maize plants at different distances to the FluorCam panels. In this way, plants with different heights but equal photosynthetic activity were simulated. In case of the small FluorCam panel a decrease in the distance of about 7 cm lead to an increase in the light intensity of about 10%, but only caused a slight decrease of about 6.5% of Φ_PSII_ in Arabidopsis plants (Fig. 3a). Similar tests were performed with tobacco and maize plants in the system for large plants, where a maximal increase of the distance to the light source by 60 cm decreased the incident light intensity by 18%. Tobacco plants were found to be affected by this increase in the distance to the light source, with an increase in values of Φ_PSII_ by about 8% (Fig. 3b). In contrast, the same increase in the distance to the light source showed almost no effect on Φ_PSII_ in maize plants (Fig. 3c). Additional tests performed with a panel of eight maize accessions validated the weak effect of the distance to the light source on the determined Φ_PSII_ values in maize plants (Fig. 4a). For a selection of four maize accessions light response curves of the electron transport rates have been recorded by repeated measurements of Φ_PSII_ and calculation of respective ETRs with increasing light intensities (up to the maximum light intensity of the large FluorCam panel of 600 µmol m^−2^ s^−1^). For all tested maize accessions, the different light intensities were found to drive photosynthetic electron transport rates in the linear part of the light response curve (Fig. 4b). This indicates that values of Φ_PSII_ as the basis for ETR calculation differed only marginally with changing light intensities and, vice versa, that differences in the incident light intensity have minor influence on the detected values of Φ_PSII_. These experiments demonstrate that the presented systems for small and large plants allow reliable and comparable Φ_PSII_ determination in Arabidopsis and maize populations with differences in plant height of up to 7 and 60 cm, respectively. For other species such as tobacco, size and developmental stage dependent effects have to be tested and need to be considered in HT CFI measurements.

### Implementation of HT-amenable protocols for the quantification of PSII properties

CFI enables the quantification of a series of different chlorophyll fluorescence parameters by the application of protocols that differ in duration and complexity (Table 1). The protocol design essentially determines the measurement time per plant (carrier) and was here optimized for selected important photosynthetic features towards a maximum throughput capacity with the given logistical opportunities of the integrated LemnaTec-FluorCam systems.

**Fig. 5:**
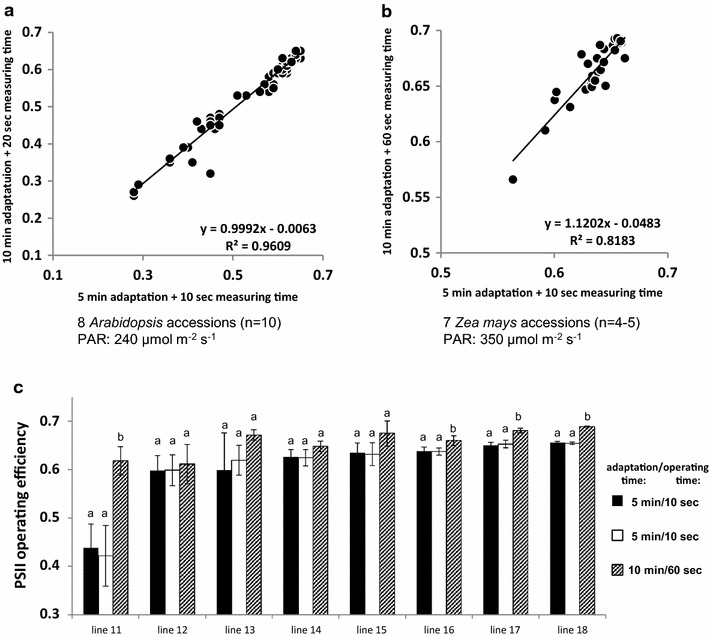
Comparison of different operation protocols for determination of Φ_PSII_ in HT performance. Protocols differ in operating times (stay in FluorCam chamber) as well as in adaptation times (stay in adaptation tunnel). **a** System for small plants. **b** System for large plants. **c** Influence of adaptation and operating time variation on the values of Φ_PSII_ measured at a PAR of 350 µmol m^−2^ s^−1^ in different maize accessions (48 DAS). Measurements were carried out successively at one day in periods of 38 or 75 min, respectively (total time 4 h). The data are the mean ± SD of 4–5 replicate measurements. *Letters* indicate statistically significant differences at the *p* < 0.05 level according to ANOVA and post-hoc Bonferroni test

**Fig. 6:**
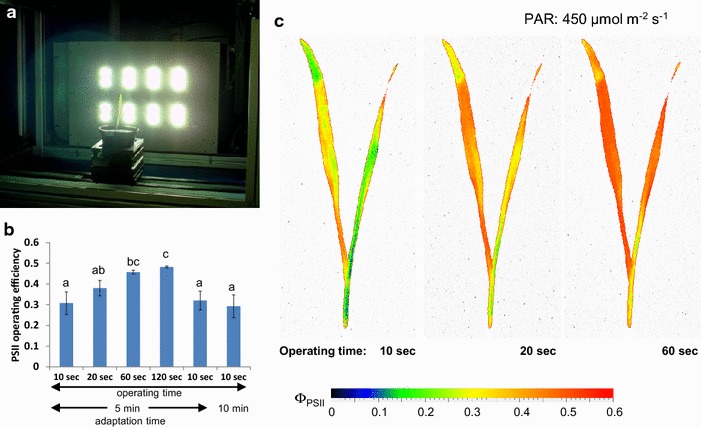
Side view operation as an additional application of FluorCam-device in system for small plants. **a** Set-up of FluorCam-module in side view operation. **b** Influence of operating and adaptation time variation on the value of Φ_PSII_ in wheat seedlings cv. “Kanzler” (9 DAS). Measurements were carried out successively at one day in periods of 9–15 min (total time 130 min). The data are the mean ± SD of 4 replicate measurements. *Letters* indicate statistically significant differences at the *p* < 0.05 level according to ANOVA and post-hoc Bonferroni test. **c** Φ_PSII_ images of a wheat seedling captured at different operating times. Measurements were performed at a PAR of 450 µmol m^−2^ s^−1^

The determination of F_v_/F_m_ relies on a relatively simple and fast measurement protocol. In the presented system for small plants, F_v_/F_m_ determination of dark-adapted plants (usually measured 2 h after onset of the dark period) can be achieved with a maximum capacity of 154 carriers per hour which corresponds to 1848 plants/hour when using the 12-well-trays supporting 12 plants per carrier (Table 3). Due to longer transport distances of carriers in the large system, a maximum capacity of 70 carriers/h and 280 plants/h (using configuration of 4 pots per carrier) can be obtained (Table 3). The determination of Φ_PSII_ requires that plants have reached a fully light-adapted state, which is realized through measurement of plants during the light phase and additional exposure to homogeneous illumination in the adaptation tunnels prior to the actual measurement process. Using a small panel of selected Arabidopsis accessions with varying photosynthetic performance, a HT-optimized measurement protocol was developed and implemented. The aim was to minimize illumination times in the adaptation area as well as during the FluorCam measurement while ensuring full light adaptation and the acquirement of precise and accurate results. The optimization procedures resulted in an experimental protocol comprising an incubation time of 5–8 min in the adaptation tunnel immediately followed by a 10 s illumination period (with equal light intensity as during adaptation) after movement into the CFI chamber (taking less than 10 s) and prior to the saturating flash. This protocol was validated by measurements with doubled durations of both illuminations, which resulted in very similar Φ_PSII_ values as revealed by a very strong positive correlation between the different protocols (Fig. 5a). Using the optimized protocol, a maximum number of 90 carriers/h (corresponding to 1080 plants/h using the 12-well-tray configuration) can be analysed in the small-plant system. For young seedlings of plant species with an upright architecture, such as monocotyledonous plants, the side view CFI modus may be more appropriate (Fig. 6a). Experiments with wheat seedlings demonstrated that changes in the adaptation time in the tunnel with illumination from the top did not affect the measured Φ_PSII_ values. On the other hand, an increase in the side-view illumination time inside the CFI chamber significantly changed the measured PSII operating efficiency (Fig. 6b, c). This indicates that determination of Φ_PSII_ through side view CFI also requires an adequate side illumination to reach a steady state of full light adaptation. Apparently for reasons of plant architecture (steep leaf angle of wheat seedling leaves) the fraction of the light intercepted from the top illumination in the adaptation tunnel is not sufficient to induce a steady level of PSII operating efficiency when this is measured through side illumination and imaging. The orientation of leaves in parallel to the plane of the FluorCam panel in side view CFI mode results in the interception of a considerably higher fraction of the incident light, triggering further changes in PSII operating efficiency. Thus, prolonged initial side illumination period is necessary in the side mode CFI procedure which extends the required operation time. As the current setup allows side view illumination only inside the CFI chamber on a single-carrier and single-plant basis, the maximum capacity for determination of Φ_PSII_ using the side-view modus is about 50 carriers/h.

**Table 3:** Capacities of the integrated systems for chlorophyll fluorescence imaging (FluorCam) according to the used protocol

Protocol	System for small plants		System for large plants	
	Carrier/h	Plants/h (12 plant trays)	Carrier/h	Plants/h (4 plant trays)
F_v_/F_m_	154	1848	70	280
Φ_PSII_ at steady state	90	1080	46	184
Light response curve 6 light intensities; 1 min each	10	120	8–9	32–36

Similar optimization experiments were conducted in the system for large plants, which allows top view (only) CFI for plants up to 1.80 m height. Also here, an HT-suitable protocol for the determination of Φ_PSII_ was established, and 5–8 min adaptation time in the tunnel and 10 s illumination in the CFI chamber were found to be sufficient to achieve highly reproducible results for all tested maize accessions (Fig. 5b, c). Extension of adaptation (10 min) and illumination time (60 s) led to very slight increases of the Φ_PSII_ value (2–7%) for seven of the eight maize accessions tested. Only for one accessions (line 11), a substantial influence of a prolonged measuring time in the imaging station (60 s) on Φ_PSII_ was found (Fig. 5c). Plants of this line were particularly small and were partly shaded by plants of neighbouring carriers during their stay in the tunnel and were therefore probably not fully light acclimated prior to the CFI measurement. In general, due to the different design of the conveyor system and the much larger transport distances, the maximum throughput capacity in the system for large plants is lower (46 carriers/h corresponding to maximum 184 plants/h using the 4-pot configuration) compared to the system for small plants (Table 3). Depending on the purpose of the experiment, ranging from screening of very large populations of plants to select candidates for further investigations to precise analyses of selected sets of plants, the protocols may be adapted to either allow maximum throughput (at the expense of some degrees of precision) or to support highly accurate analyses (e.g. using extended adaptation and illumination times) for reduced numbers of plants.

The above described HT optimized protocols enable the fast and dynamic screening of large model and crop plant populations for F_v_/F_m_ and Φ_PSII_. The FluorCam modules additionally offer the possibility to measure further photosynthetic parameters via more complex procedures such as light response curves with minute-wise decreasing or increasing light intensities or complete quenching protocols (NPQ, q_L_). Due to longer measurement times (of several minutes), such measurements can be performed in medium to low throughput (8–10 carriers/h), e.g. for candidate genotypes/accessions pre-selected according to certain F_v_/F_m_ and Φ_PSII_ values. Further experiments will be necessary in order to validate measurements of NPQ and other fluorescence parameters.

### Validation of HT CFI fluorescence measurements

**Fig. 7:**
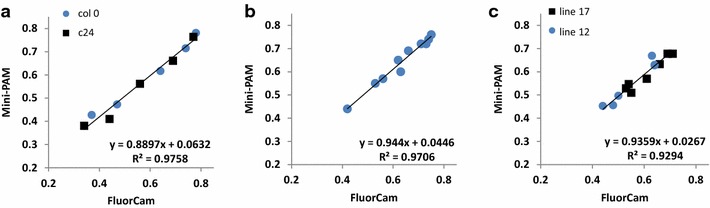
Relationship between values of Φ_PSII_ measured using the FluorCam-system and values obtained with the non-imaging chlorophyll fluorimeter Mini-PAM. **a** System for small plants operating in top view orientation (Arabidopsis accessions, 19 DAS). **b** System for small plants operating in side view orientation (wheat seedlings, 9 DAS). **c** System for large plants operating in top view orientation (maize accessions, 37 DAS). In the case of the system for small plants, the actinic light intensities were varied between 12 and 590 µmol m^−2^ s^−1^ and in the case of the system for large plants between in 138 and 600 µmol m^−2^ s^−1^

In order to validate the Φ_PSII_ data acquired with the automated HT CFI system, we compared the whole-shoot image-based mean values with manual measurements using the low throughput spot-measuring Mini-PAM fluorometer (Heinz Walz GmbH, Effeltrich, Germany) equipped with the leaf-clip holder 2060-B (small plant system) or 2030-B (large plant system). CFI derived data acquired for Arabidopsis and wheat seedlings from the top and side view in the small plant system, and of maize plants from the large plant system were compared with manual measurements of fully developed Arabidopsis leaves (Fig. 7a), or spot measurements in central areas of young wheat leaves (Fig. 7b) and fully developed maize leaves (Fig. 7c). Mini-PAM measurements were performed immediately after (Arabidopsis and wheat) or before (maize) the CFI. For all plants tested, significant positive correlations (R^2^ > 0.92) between manual and automated image-based Φ_PSII_ measurements were detected (Fig. 7). The very strong correlations between whole-shoot and spot measurements in different species confirm the relevance and accuracy of the CFI measurements acquired in the presented integrated setup.

### Screening of large Arabidopsis and maize accession panels for variation in PSII operating efficiency

**Fig. 8:**
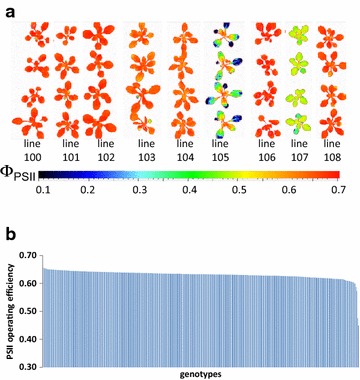
Screening of 384 Arabidopsis accessions for alterations in Φ_PSII_ using the system for small plants. **a** Examples of images of Φ_PSII_ captured for interesting Arabidopsis accessions. **b** Scheme illustrates variation of Φ_PSII_ among investigated Arabidopsis accessions (19 DAS). The data are the means of Φ_PSII_ values of 5–12 plants. Measurements were performed at a PAR of 240 µmol m^−2^ s^−1^

**Fig. 9:**
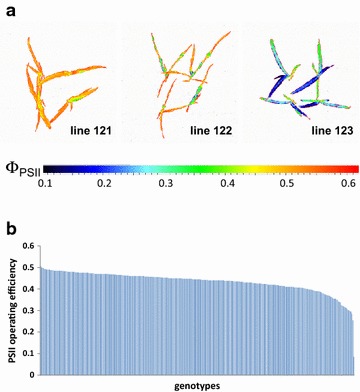
Screening of 326 maize accessions for alterations in Φ_PSII_ using the system for large plants. **a** Examples of images of Φ_PSII_ captured for representative maize accessions. **b** Scheme illustrates variation of Φ_PSII_ among investigated maize accessions (26 DAS). The data are the means of Φ_PSII_ values of 1–8 plants. Measurements were performed at a PAR of 600 µmol m^−2^ s^−1^

With the aim of proving the HT applicability of the systems, panels of model (Arabidopsis) and crop (maize) plants comprising 384 and 326 accessions, respectively, were screened for variation in Φ_PSII_ using the HT-optimized measurement protocols (5 min light adaptation, 10 s actinic illumination during the measurement). For Arabidopsis, the measurement was performed on seedlings at 19 days after sowing (DAS) with up to 12 replicates per accession (3 groups of four plants) randomly distributed throughout the phytochamber of the system for small plants. Among the tested Arabidopsis accessions, two showed particularly low Φ_PSII_ levels of 0.45 and 0.48 (Fig. 8a). Moreover, line 105 displayed pronounced spatial variation in Φ_PSII_ among different leaves. All other Arabidopsis accessions showed only minor variation in Φ_PSII_ ranging gradually from 0.57 to 0.66 (Fig. 8b). Analysis of the 326 maize accessions was performed in the system for large plants. Up to eight replicates per accession (four per carrier) were monitored. The data revealed a much higher variation in Φ_PSII_ among maize accessions compared to Arabidopsis (Fig. 9a) with whole plant mean values in the range of 0.255–0.505 (Fig. 9b). The observed differences among selected subsets of Arabidopsis and maize accessions were reproduced with independently cultivated and measured plants (data not shown). These results demonstrate the applicability of the implemented HT-optimized CFI protocols for the analysis of large plant populations for variation in Φ_PSII_.

## Discussion

The present study describes the integration of PAM-based imaging systems for functional chlorophyll fluorescence into existing HT plant phenotyping facilities set up for non-invasive and dynamic quantification of plant architecture- and biomass-related traits. These upgraded facilities thus enable the integrated assessment of plant structural and physiological features of large numbers of model and crop plants up to 1.80 meters height in very short time intervals. High throughput suitable protocols for the measurement of relevant chlorophyll fluorescence parameters (F_v_/F_m_, Φ_PSII_) have been implemented, validated and applied for screening Arabidopsis and maize populations in order to assess the extent of natural variation in photosynthetic efficiency.

The first HT CFI systems have been set up already some years ago with a focus on the simultaneous screening of many small seedlings, especially Arabidopsis or other rosette plants [13, 18–20]. These platforms mostly work according to the sensor-to-plant principle with a module containing the imaging unit and light sources moving over a stationary multi-well planting tray. In contrast, the platforms described herein consist of a stationary imaging/lighting module which measures plants that are transported into respective imaging stations in special transport carriers on conveyor belts. This has the advantage that the number of plants that can be screened is only limited by the growing area. Moreover, the FluorCam panel is vertically adjustable and allows the measurement of plants with various heights. Similar integrated plant-to-sensor systems have been established more recently which combine CFI with other imaging approaches (mostly RGB, partially HyperSpec). Platforms such as the National Plant Phenotyping Infrastructure (NaPPI, Helsinki, Finland, http://blogs.helsinki.fi/nappi-blog/highthroughput/), the Crop Plant Shoot Module at the High Resolution Plant Phenotyping Centre (HRPPC, Canberra, Australia, http://www.plantphenomics.org.au/services/cropshoot/) and several PlantScreen™ phenotyping systems (at DGIST, Daegu, South Korea; at the Center of the Region Haná for Biotechnological and Agricultural Research, China; Olomouc, Czech Republic and at IBERS, Aberystwyth, UK) apply the CFI measurements using modulated light for the HT throughput analysis of small plants (such as Arabidopsis) and/or low to medium throughput for medium sized plants (such as grasses, legumes, rice with a plant height up to 1.40 m). Another installation at the Bellwether Foundation Phenotyping Facility (St. Louis, USA, https://www.danforthcenter.org/scientists-research/core-technologies/phenotyping) enables fast Kautsky-type CFI measurements for small to medium sized plants.

The here presented integrated CFI system for small plants is able to achieve a throughput of about 1080 plants per hour for measurements of Φ_PSII_. This is comparable to the capacity of the Phenovator system at the Wageningen University, The Netherlands (approximately 1440 plants/h [20]). For the determination of the maximum PSII efficiency in dark-adapted plants (F_v_/F_m_) our system can handle about 1848 plants/h which represents a substantial increase in capacity compared to other systems (Phenovator [20], GROWSCREEN FLUORO [19]). However none of the abovementioned platforms allows the CFI-based analysis of plants taller than 1.40 m. We present here a CFI system and HT-suitable screening protocols which allow the accurate measurement of photosynthetic parameters in large plants up to 1.80 m height with a maximum capacity of about 184 and 280 plants/h for Φ_PSII_ and F_v_/F_m_ measurements, respectively. The presented setup enables the relatively fast automated measurement of hundreds of plants, which should be sufficient for instance for the assessment of the diurnal dynamics of photosynthetic parameters and may furthermore enable the implementation of more complex experimental protocols. For precise and accurate measurements of photosynthetic parameters it is an important prerequisite that plants have reached the fully dark or light-adapted states prior to the measurements. In the presented setup, the latter is ensured by light treatment with large LED panels providing homogeneous actinic illumination in the light adaptation tunnel and imaging station. In the system for large plants we work with relatively large illuminated adaptation/imaging areas and substantial height ranges. Using the special design of a high density of focussed LEDs, only small differences in the incident actinic light intensities occurred with increasing distance of the light source, which did not substantially affect the quantification of PSII operating efficiency in maize plants. Potential effects due to differences in the (horizontal) orientation of leaves relative to the light source and shading effects were found to be largely negligible or only rarely occurring for maize (and completely avoided in the system for small plants). This was furthermore supported by a very good correspondence between HT CFI data (Φ_PSII_) and data of spot measurements performed with a standard protocol using a Mini-PAM fluorimeter. These findings give evidence for the validity of the HT CFI method as a screening approach for large numbers of plants. Nevertheless, according to the observation of potentially occurring unintended environmental effects (e.g. through very large neighbouring plants on smaller individuals as in the case of maize) or the potential slight effects of extended adaptation/illumination times, it is generally recommended, and potentially necessary in some cases, to carry out iterative analysis regimes involving re-analysis of subsets of individuals after an initial broad screen or to verify outliers through additional in-depth analyses in order to distinguish biological from rarely occurring methodological variation. The non-invasive nature of CFI and the achievable run times of the platforms allow re-analysis of the very same individuals within hourly or daily intervals and thus are well suited to carry out such series of connected experiments.

The described integration of CFI panels into LemnaTec Scanalyzer systems enables the integration of CFI data with a huge number of plant traits derived from LemnaTec sensors (for NIR/RGB and static FLUO imaging) through image analysis routines implemented in the integrated analysis platform (IAP) framework [37, 38]. The IAP-based feature extraction allows the quantification of ~200 plant traits in different categories such as biomass-related traits (projected leaf areas, volumes), morphological/architectural traits, colour-related and intensity based traits from near infrared and static fluorescence imaging which have been shown to be relevant for the description of growth dynamics, architecture and stress response in various plant species (Arabidopsis: [4, 39], maize: [4, 40], rice: [41], lentils: [42], barley: [3, 43]). The integration of IAP-based parameters such as plant height and width (as proxies for plant erectness), compactness and coloration (greenness) with CFI-based chlorophyll fluorescence parameters is of special relevance for the analysis of relations between plant growth and architecture and photosynthetic processes. Current efforts in IAP developments include the tracking and segmentation of individual organs such as leaves or flowers (unpublished) which will enable the gain of additional information about the spatial heterogeneity, beyond the determination of a whole-plant mean phenotypic parameter value. The organ-based analysis will be further enhanced and supported by the integration of 3D data which enables the 2D/3D co-registration-based enrichment of 2D images with height profiles of plants (mapping of photosynthetic functional properties onto single leaves in 3D). 3D laser scanners have recently been installed in the phenotyping systems for small and large plants and will allow the quantification of novel 2D/3D integrated traits (such as PSII performance in the context of leaf angular distribution). An accurate description of plant architecture is inevitable when trying to quantify the biomass gain from photosynthesis [5]. Thus photosynthetic engineering is essentially based on the fundamental knowledge gained from the integrated analysis of plant architecture and physiology. In that respect the availability of corresponding HT phenomics facilities and respective HT-optimized protocols as the one(s) described herein are of utmost importance as they build the basis for the quantitative, non-invasive and dynamic assessment of integrative plant traits combining information about plant architecture and physiology. In this context the herein described uses of the systems for screening of natural variation in photosynthetic performance in large populations of Arabidopsis and maize represent the first steps towards assessing the potential of genetic variation in photosynthesis-related yield components [22]. Selected candidate accessions with alterations in photosynthetic performance will be subject of further detailed investigations on the physiological and architectural level using the presented phenotyping platform and its future upgrades. In further genetic studies HT CFI derived physiological parameters will serve as a quantitative trait for genetic mapping towards identification of the causal genes and their allelic variation and towards marker assisted breeding for improved crop productivity.

## Conclusions

The described incorporation of CFI systems into existing automated HT phenotyping platforms substantially extends the feature spectrum to be extracted through the simultaneous assessment of plant architectural and biomass-related traits and its relations to physiological features such as PSII operating efficiency. The reliability of the method in a HT performance was verified by comparative non-imaging fluorescence measurements. We demonstrated that the implemented HT protocols are applicable to a broad spectrum of model and crop plants of different sizes (up to 1.80 m height) and architectures. This enables researchers to gain a deeper understanding of the relation of plant architecture, biomass formation and photosynthetic efficiency based on advanced HT phenotyping methods which bears a great potential with respect to crop and yield improvement strategies.

## Methods

### Plant material and cultivation

Various accessions of *Arabidopsis thaliana* (L.) and plants of *Triticum aestivum* (L.) cv. “Kanzler” were grown under controlled conditions at 20/18 °C, 60/75% relative humidity, PAR of 250 µmol m^−2^ s^−1^ from Whitelux Plus metal halide lamps (Venture Lighting Europe Ltd., Rickmansworth, Hertfordshire, England) and under a 16/8 h day/night regime in a walk-in growth chamber. Plants were grown in a mixture of 85% (v) red substrate 1 (Klasmann-Deilmann GmbH, Geeste, Germany) and 15% (v) sand.

Seeds of the analysed Arabidopsis accessions were bulk-amplified from stocks homogenised by single-seed propagation [44]. These accessions were selected to represent a large portion of the genetic variation as captured in the 250K SNP chip [45]. All seeds used for the experiments described here were amplified simultaneously in a greenhouse under long-day conditions in 2012.

*Zea mays* and *Nicotiana tabacum (SNN)* plants were grown in a climate controlled glass house at 25/20 °C day/night, 65% relative humidity and supplemental illumination using SonT Agro high pressure sodium lamps (Philips, Amsterdam, The Netherlands) achieving a PAR of 350 µmol m^−2^ s^−1^ with a light period of 16 h. The maize seedlings were pre-cultured in small pots (9 cm diameter) filled with substrate 2 (Klasmann-Deilmann GmbH, Geeste, Germany) for 5 days. Thereafter the plants were grown in a mixture of 40% IPK self-made compost, 40% substrate 2 and 20% sand [4]. The same mixture was used for the cultivation of tobacco plants. For screening experiment *Z. mays* seeds were germinated in 6-well trays filled with substrate 2 after 24 h imbibition and cultivated in a walk-in growth chamber at 15/10 °C day/night, 65% relative humidity and PAR of 250 µmol m^−2^ s^−1^ from Whitelux Plus metal halide lamps with a light period of 16 h for 10 days. Thereafter the seedlings were transplanted into 5 L pots filled with a mixture of 40% IPK self-made compost, 40% substrate 2 and 20% sand and grown in a climate controlled glass house at 10/15°C day/night, 65% relative humidity and supplemental illumination using SonT Agro high pressure sodium lamps achieving a PAR of 350 µmol m^−2^ s^−1^ for further 10 days followed by a 14 day period at 20/17 °C day/night and a final growth temperature of 25/20 °C day/night (all other parameters were kept unchanged).

Seeds of the analysed maize accessions were derived from the Genebank of the Leibniz Institute of Plant Genetics and Crop Plant Research (IPK) or from a maize diversity panel [40] and represent accessions with a wide variation in root and shoot traits.

### Measurements of PAR intensity

PAR measurements were performed with a quantum sensor (PAR lite Meteon, Kipp&Zonen, Reichenbach, Germany) placed in the centre of the carrier or light panel, respectively. Homogeneity of PAR was verified by multiple measurements over the top of the carrier area (17 measuring points per 2210 cm^2^ in case of the system for large size plants and 5 measuring points per 150 cm^2^ in case of the system for small size plants, respectively). The distribution of measuring points across the carrier top area is illustrated in Fig. 2c–e.

### Chlorophyll fluorescence measurements (Mini-PAM)

Chlorophyll a fluorescence measurements were performed with a Mini-PAM fluorometer (Heinz Walz GmBH, Effeltrich, Germany) equipped with a leaf clip holder either 2060-B (system for small size plants) or alternatively 2030-B (measurements on maize plants). Φ_PSII_ was calculated as F_q_′/F_m_′ where F_q_ is the difference in fluorescence between F′ (steady state fluorescence of the light-adapted probe) and Fm′, the maximum fluorescence yield in the light-adapted state induced by the application of a saturating light pulse with a duration of 800 ms (white light, intensity of circ. 7000 µmol m^−2^ s^−1^). Actinic light was provided by the internal halogen lamp of the device using the fibre-optics (duration 10 s) and intensity was varied between 50 and 595 µmol m^−2^ s^−1^ (white light). Light intensities were measured by the microquantum sensor of the Mini-PAM. Measurements were performed with plants light-adapted in adaptation tunnel for 10 min immediately after (system for small size plants) or before (system for large size plants) imaging of the plants took place using the FluorCam-system. The same light intensity was used during adaptation in the tunnel and in the imaging chamber.

### Chlorophyll fluorescence imaging (FluorCam)

Imaging of chlorophyll a fluorescence was performed using FluorCam imaging fluorimeters (Photon Systems Instruments, Brno, Czech Republic). Shutter time and sensitivity of the charge-coupled device (CCD) camera (SN_FC800) were adapted to the particular object. Measurements of Φ_PSII_ (see above) were made after light adaptation of the plants in adaptation tunnel and subsequent to an illumination period in the FluorCam-chamber as indicated in the results section. Duration of the saturating light pulse to induce F_m_′ was 800 ms with an intensity of 4100 µmol m^−2^ s^−1^ (white light) in the system for small plants and 3800 µmol m^−2^ s^−1^ in the large system, respectively. Light response curves of the photosynthetic electron transport of PSII (ETR) were obtained by a stepwise decrease of the actinic light intensity (duration of each step 60 s). The apparent rate of ETR was determined as ETR=Φ_PSII_ × PAR × 0.5 × 0.84 where 0.5 is a factor that accounts for the fraction of excitation energy distributed to PSII and the factor 0.84 corresponds to the leaf absorbance. Both factors are empirical mean factors. Therefore the results of the ETR calculations are considered as apparent.

## Abbreviations

CCD: charge coupled device
DAS: days after sowing
ETR: apparent rate of photosynthetic electron transport of PSII
F_v_/F_m_: dark-adapted (maximum) PSII efficiency
HT: high-throughput
IAP: integrated analysis platform
LED: light emitting diodes
NPQ: non-photochemical quenching coefficient
PAM: pulse amplitude modulation
PAR: photosynthetic active radiation
PSII: photosystem II
Q_A_: plastochinone A
Φ_PSII_: PSII operating efficiency
QTL: quantitative trait loci
SD: standard deviation
